# Estimated herd prevalence and sequence types of *Coxiella burnetii* in bulk tank milk samples from commercial dairies in Indiana

**DOI:** 10.1186/s12917-015-0517-3

**Published:** 2015-08-07

**Authors:** Amy E. Bauer, Sonora Olivas, Maria Cooper, Heidie Hornstra, Paul Keim, Talima Pearson, April J. Johnson

**Affiliations:** Department of Comparative Pathobiology, College of Veterinary Medicine, Purdue University, West Lafayette, IN USA; The Center for Microbial Genetics and Genomics, Northern Arizona University, Flagstaff, AZ USA; Indiana State Board of Animal Health, Indianapolis, IN USA; Department of Comparative Pathobiology, Purdue University, West Lafayette, IN USA; Present address: Signature Science, LLC, Baku, Azerbaijan

## Abstract

**Background:**

*Coxiella burnetii* is the etiologic agent of Q fever, a zoonotic disease causing influenza-like illness, pregnancy loss, cardiovascular disease and chronic fatigue syndrome in people. *C. burnetii* is considered to be enzootic in ruminants, but clinical signs of infection do not always manifest. National studies have documented the presence of *C. burnetii* in dairy herds in Indiana. This represents an opportunity to better characterize the distribution and prevalence of *C. burnetii* infection at the state scale, allowing evaluation of the need for surveillance and response planning to occur at this level. A cross-sectional study was conducted to estimate the herd prevalence of *C. burnetii* in commercial cattle dairies in Indiana and characterize the strains of *C. burnetii* within these dairies.

**Results:**

Bulk tank milk samples were collected between June and August of 2011 by the Indiana State Board of Animal Health (ISBOAH). A total of 316 of these samples were tested for the *IS1111* transposon of *C. burnetii* using quantitative real time polymerase chain reaction (PCR). Single nucleotide polymorphism (SNP) genotyping was used to identify the multispacer sequence genotypes (ST) present in samples where the *IS1111* transposon was identified. The geographic distribution of dairies testing positive for *C. burnetii* DNA and the identified STs were also evaluated. The estimated overall herd prevalence for *C. burnetii* DNA was 61.1 % (95 % CI 55.6–66.3 %). The highest estimated regional prevalence was 70.2 % in the Central region of Indiana. An ST was identifiable in 74 of the positive 178 samples (41.6 %) and none of the 10 negative samples tested. Of these samples, 71 (95.9 %) were identified as *ST20*, 2 (2.7 %) as *ST8* and a combination of *ST20* and *ST8* was identified in a single sample.

**Conclusions:**

*C. burnetii* is present in dairy herds throughout Indiana. Indiana follows national trends with *ST20* most commonly identified. The presence of multiple STs in a single bulk tank sample indicates that multiple strains of *C. burnetii* can circulate within a herd. This supports potential transmission of *C. burnetii* between goats and cattle, presenting the potential for a switch in the dominant genotype found in a given species.

**Electronic supplementary material:**

The online version of this article (doi:10.1186/s12917-015-0517-3) contains supplementary material, which is available to authorized users.

## Background

*Coxiella burnetii*, a gram-negative, intracellular bacterium, is the causative agent of the zoonosis Q fever, which was first described among abattoir workers in Australia [1]. *C. burnetii* is now considered to be present throughout most of the world including the United States. Although *C. burnetii* is capable of infecting many animal species, ruminants (cattle, sheep, and goats) are considered to be the main reservoir for human infection [2, 3]. The most common route of transmission is through inhalation of aerosolized particles. *C. burnetii* may be carried by wind for at least 2 km thus direct contact with ruminants, facilities where ruminants are housed and cared for, or ruminant products is not necessary for transmission [4–6]. Easy aerosolization, low infectious doses, and high transmissibility have contributed to *C. burnetii*’s classification as a Class B bioterror agent [7].

While infection with *C. burnetii* is considered enzootic in domestic ruminants, there is often no clinical disease and infection may go undetected in these species [3]. When clinical signs manifest, reproductive effects such as abortion, stillbirth or weak offspring are most commonly seen [7–9]. Clinical signs can vary by species and small ruminants are more likely to demonstrate abortion while cattle are more likely to exhibit infertility or metritis [10]. *C. burnetii* is present in great quantities in the placenta and reproductive discharges at the time of abortion or parturition [8]. It can also be shed in milk, fecal material, vaginal secretions and urine [9, 10] leading to environmental contamination. Once shed into the environment, *C. burnetii* is resistant to desiccation and extreme temperatures, enabling it to persist for weeks to months where contamination has occurred [3].

Bulk tank milk analysis [either by PCR or enzyme-linked immunosorbent assay (ELISA)] has been used to screen for *C. burnetii* in cattle, sheep, goats and camels in several countries with results ranging from 0 to 94 % prevalence depending upon country, species and analytic method used [11–16]. In a study of bulk tank milk samples from throughout the United States, including Indiana, more than 90 % of the herds sampled were positive for *C. burnetii* DNA by PCR [11]. In a different study, the National Animal Health Monitoring Systems (NAHMS) evaluated 528 dairies that provided a single sample of bulk tank milk for testing for *C. burnetii* DNA [17]. Indiana was also represented in this sample. The overall herd level prevalence estimate in this study was 77 % [17].

Multiple genetic methods have been developed to identify different strains of *C. burnetii*, including restriction fragment length polymorphism (RFLP) [18], multilocus variability analysis (MLVA) [19, 20], and multispacer sequence typing (MST) [21]. Focusing upon 10 spacers identified by MST, 34 sequence types (ST) of *C. burnetii* have been identified [21]. A rapid genotyping method was developed based upon analysis of single nucleotide polymorphisms (SNPs) within these STs [22]. Use of this SNP analysis led to the identification of 2 STs that show a high degree of host specificity in commercial milk samples in the United States, *ST20* in cattle milk and *ST8* in goat milk [23].

Although human Q fever has been related to consumption of unpasteurized milk [24], recent outbreaks have been associated with goats rather than cattle [6, 25]. While there is currently debate as to the role of dairy products as sources of *C. burnetii* infection in people [26, 27], cattle may still serve as a source of environmental contamination and human infection through reproductive materials. In 2012, cattle were a more prominent commodity in the United States than small ruminants (89,994,614 cattle and calves as compared to 2,621,514 goats and kids) [28]. In 2012 there were an estimated 174,141 dairy cattle in Indiana [29], whereas the population of goats in Indiana was 38,632 [30]. The role of goats as a reservoir for *C. burnetii* in Indiana should not be neglected, but the larger population of cattle carries with it a potential for greater levels of human contact with this reservoir species and its products. Estimating the prevalence of *C. burnetii* and its genotypes in cattle at the state level will allow comparison with these measures in other species, including goats and humans. These comparisons could help to develop a more complete picture of the ecology, epidemiology and transmission dynamics of *C. burnetii*.

Samples from Indiana have been included in national studies [11, 17, 23], but the prevalence of *C. burnetii* in commercial dairy herds at the state level as well as the ST of *C. burnetii* present within these herds have not been previously evaluated. Documentation of the prevalence of *C. burnetii* in 2011 creates a baseline for future reference in animal outbreaks of Q fever in Indiana, will help to trace the sources of future outbreaks and will allow for comparison with *C. burnetii* prevalence and strains present in other species and geographic locations during this time period. In order to estimate the herd prevalence of *C. burnetii* in Indiana, a cross-sectional study was conducted focusing on bulk tank milk samples from commercial dairy cattle herds throughout the state. Samples testing positive for the presence of *C. burnetii* were genotyped using SNP evaluation to identify the strain or strains present in that herd.

## Methods

### Samples and sample size calculation

**Fig. 1:**
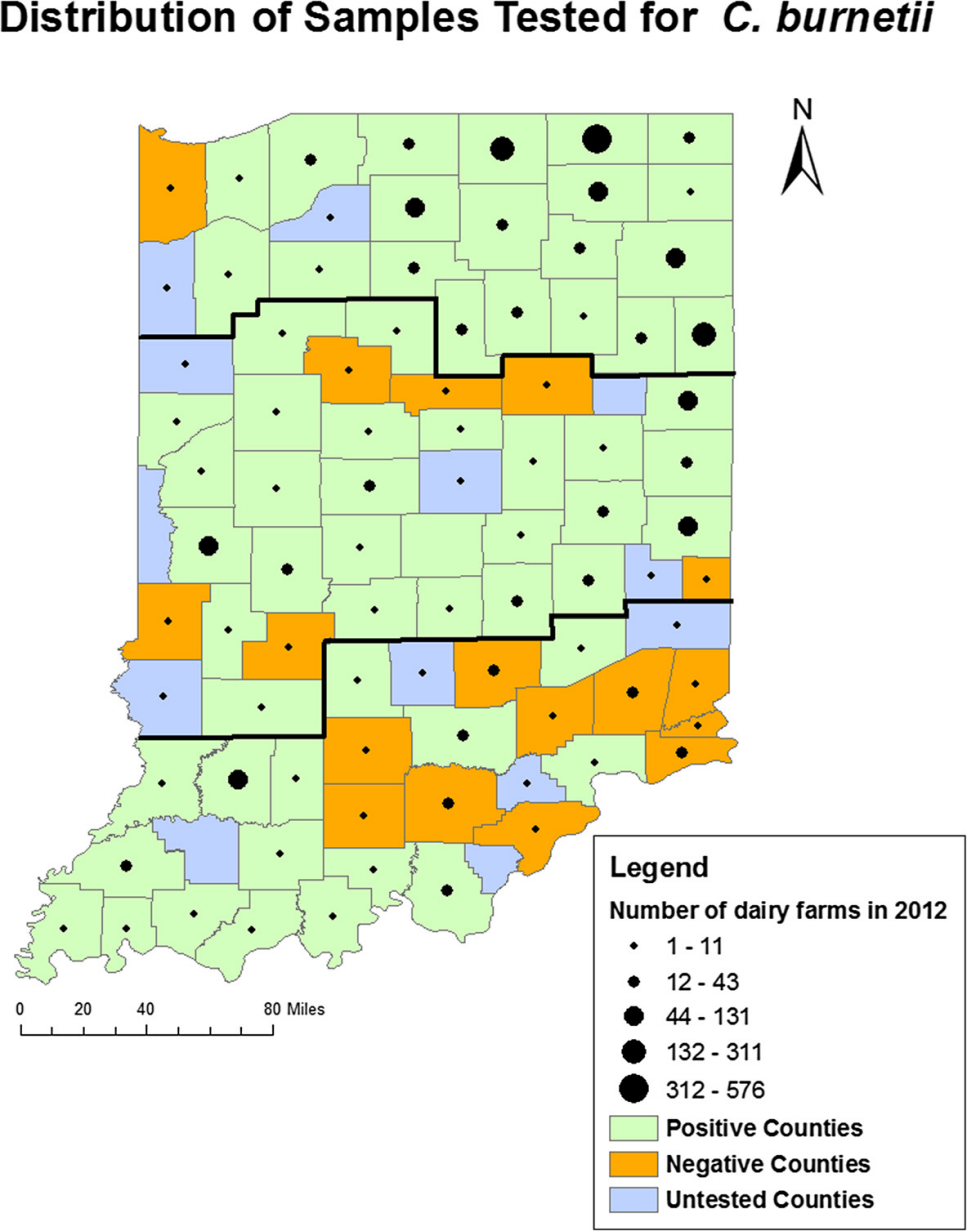
Geographic distribution of samples tested for *C. burnetii.* Indiana counties with bulk tank milk samples tested for *C. burnetii*. Counties with bulk tank milk samples that tested positive for *C. burnetii* are coded green. Thick lines indicate the regional boundaries that were defined by Public Health Preparedness Districts. Dots indicate the number of dairy herds per county in 2012 as reported by the USDA National Census of Agriculture. Counties without dots were not reported as individual counties but included in the overall total of 2401 dairy herds

The United States Department of Agriculture 2012 Census of Agriculture identified 2401 premises in Indiana with dairy cattle [29]. The preponderance of these premises were located in the Northern region of Indiana (Fig. 1 and Table 1). Ninety percent of these farms (*n* = 2151) had fewer than 100 cattle in the herd with 901 farms (37.5 %) having a herd size of fewer than 10 cattle [29]. Although the census did not specifically differentiate between dairies engaged in commercial production of milk and other premises where dairy cattle were housed, estimates from the ISBAOH indicated that between 1200 and 1225 premises were producing milk commercially in 2011 (personal communication).

**Table 1:** Geographic distribution of dairy herds and estimated herd prevalence of *C. burnetii*

Region	Total regional herds^a^	%Total herds	Total samples	%Total samples	Samples tested	%Samples tested	Positive samples	% Positive samples	Estimated prevalence	95 % C.I.
North	1622	67.6 %	845	72.4 %	217	68.7 %	129	66.8 %	59.4 %	52.8–65.8 %
Central	408	17.0 %	173	14.8 %	57	18.0 %	40	20.7 %	70.1 %	57.4–81.0 %
South	371	15.4 %	149	12.8 %	42	13.3 %	24	12.4 %	57.1 %	42.0–71.4 %
Total	2401		1167		316		193		60.8 %	55.3–66.0 %

^a^Total Regional Herds obtained from the 2012 Census of Agriculture [37]. These numbers include, but are not exclusive to, commercial dairy herds

A total of 1167 samples of bulk tank milk identified by the county of origin were obtained from the Indiana State Board of Animal Health (ISBOAH). These samples were collected from commercial dairies in Indiana in the summer of 2011 as part of three-pronged surveillance program for brucellosis in commercial dairy cattle and stored at −20 °C before and after transfer to Purdue University. The goal for the surveillance program was to obtain samples from 95 % of the estimated 1225 commercial dairies in Indiana. Records kept during sample collection indicated that a total of 1385 bulk tank milk samples were collected by the ISBAOH. To calculate sample size for estimation of herd prevalence of *C. burnetii* in Indiana, we conservatively estimated the prevalence of *C. burnetii* in the samples to be 50 %. With a 5 % allowed error and 95 % confidence, a minimum of 300 bulk tank samples were needed. Samples were stratified by county based upon the number of bulk tank samples obtained per county. The number of milk samples to be tested per county was calculated using a probability proportional to size design such that counties represented by a greater percentage of samples in the initial 1385 would be represented in greater numbers in the group screened for *C. burnetii*. For example, a county with 25 samples would make up 1.8 % of the total samples collected. With a target of 300 samples to test, 1.8 % of the 300 samples, or 5 samples from this county would be included in the study. Of the 1385 samples, 1167 (84.3 %) were provided to Purdue University and the stratification calculations were repeated based on the number of samples received. Individual samples within these counties were selected for inclusion by use of a random number generator. Counties with only 1 or 2 samples would have been excluded based on this design, but 1 sample from each of these counties was included for a more accurate representation of the distribution of herds in Indiana.

### DNA extraction and PCR

Milk samples were thawed overnight in a refrigerator or set at room temperature for 2 h prior to homogenization. DNA extraction was performed on 200 μl of homogenized milk through use of a commercial kit (Qiagen DNeasy kit, Valencia, CA) following the manufacturer’s instructions. An 86 base pair (bp) portion from bp1241 through bp1326 of the *IS1111* transposon of *C. burnetii* was amplified and detected by quantitative real time PCR using the following primer-pair and probe sequences: forward primer GAT AGC CCG ATA AGC ATC AAC, reverse primer GCA TTC GTA TAT CCG GCA TC, probe FAM-TCA TCA AGG CAC CAA T-MGBNFQ [31]. The reaction mix for each sample contained 2.5 μl 10X PCR reaction buffer, 2.0 μl 50mMol MgCl_2_, 0.5 μl 10 mM dNTPs, 2.0 μl of the 10 μM forward primer, 2.0 μl of the 10 μM reverse primer, 0.75 μl of the 10 μM probe, 0.1 μl of Platinum Taq polymerase (Applied Biosystems, Foster City, CA) and 10.15 μl of DNase, RNase free water to which 5 μl of the sample DNA was added. Negative and positive control samples were run with each PCR. Using a Stratagene Mx3000P thermocycler (Agilent Technologies, Santa Clara, CA) the sample plate was heated to 95 °C and kept at that temperature for 10 min followed by 45 cycles of 15 s at 95 °C and 1 min at 60 °C prior to analysis using MxPro (Agilent Technologies, Santa Clara, CA).

A plasmid incorporating the target DNA sequence was constructed at Purdue University in May of 2014 using the pGEM t-vector II system (Promega Life Science, Madison, Wisconsin, USA). Serial dilutions from 1×10^9^ copies to a single copy of the sequence were used to establish a standard curve. PCR was run on five replicates of each dilution to establish a measure of intra-assay variation and single replicates of each dilution were run on 5 consecutive days to establish a measure of inter-assay variation. Based on these replications, analytic sensitivity for this PCR was determined to be 100 copies of the target sequence per microliter. The average cycle threshold value for this level of detection was 36.59 on intra-assay evaluation and 35.96 on inter-assay evaluation. Samples with a cycle threshold (CT) value less than or equal to 36.5 were considered positive for purposes of analysis. Specificity analysis was not performed as part of this study. Based on the results of specificity analyses in the literature [32] and BLAST [33] searches focusing on the bp1241 – bp1326 region of the *IS1111* transposon which failed to identify organisms other than *C. burnetii* with the specific targeted sequence, the specificity of the PCR was defined as 100 % for purposes of prevalence analysis.

### Statistical analysis

Statewide and regional herd prevalence for shedding of *C. burnetii* was estimated as the number of positive herds/total number of herds tested with 95 % CI calculated. Samples were mapped by county and region based upon the Indiana State Public Health Preparedness Districts (PHPD) used by the Indiana State Departments of Health and Homeland Security, and ISBOAH. Three regions were evaluated (North = 23 counties, Central = 38 counties and South = 31 counties, Fig. 1). Using Open Epi 3.03 [34], a chi-square test of association was used to evaluate the relationship between herd shedding of *C. burnetii* DNA and geographic region. An odds ratio (OR) with a 95 % confidence interval was also assessed for this relationship using conditional maximum likelihood estimate of odds ratio.

### Sequence typing

DNA samples positive for *C. burnetii IS1111* were tested for sequence type (ST). TaqMan assays for single nucleotide polymorphisms at the *Cox56bp10*, *Cox51bp67*, *Cox22bp91* and *Cox57bp327* loci were performed [22]. The reaction mix for each sample contained 5 μl 2x TaqMan Universal PCR Master Mix (Life Technologies, CA, USA; p/n 4304437), 0.45 μl of each primer, 0.1 μl of each probe and 2.9 μl of sterile nuclease-free water to which 1 μl of DNA was added for a total reaction volume of 10 μl. Reaction conditions using an Applied Biosystems 7900HT Fast real-time PCR system were 50 °C for 2 min, 95 °C for 10 min, 45 cycles of 95 °C for 15 s and 60 °C for 1 min. Sequence Detection System v2.4 software (Life Technologies, CA, USA) was used for SNP genotyping and therefore ST assignment.

## Results

A total of 316 samples were selected for detection of *C. burnetii* DNA. Additional file 1 includes the sampling selection calculations for each county. Jasper County was initially reported to have only a single sample, however 13 samples were received from the ISBAOH and this value was used to calculate the number of samples to be tested. Selected samples represented 79 of the 92 counties in Indiana (Fig. 1). The majority of samples analyzed were from the Northern region of Indiana (Table 1). Four counties within this region (Lagrange, Elkhart, Adams and Marshall counties) accounted for the greatest number of samples tested (146, 46.1 %).

### PCR testing and prevalence calculation

Bulk tank milk samples representing 193 herds were positive for the presence of *C. burnetii* DNA by real time quantitative PCR with a cycle threshold of 36.5 or less. Based on this sample, the estimated herd prevalence for *C. burnetii* in Indiana is 61.1 % with a 95 % confidence interval (CI) of 55.6–66.3 %.

### Geographic distribution of positive herds

Herds positive for shedding of *C. burnetii* DNA were identified in all of the geographic regions of Indiana (Fig. 1). When broken into the defined regions (Table 1), the estimated herd prevalence is highest in the Central region of Indiana (70.2 %, 95 % CI 57.4–81.0 %) and lowest in the Southern region (57.1 %, 95 % CI 42.0–71.4 %). Neither Chi-square analysis (*p* = 0.29) nor odds ratios utilizing the Southern region for comparison indicated a statistically significant relationship between the geographic region of Indiana and the probability that a herd would test positive for shedding of *C. burnetii.*

### Sequence typing

**Fig. 2:**
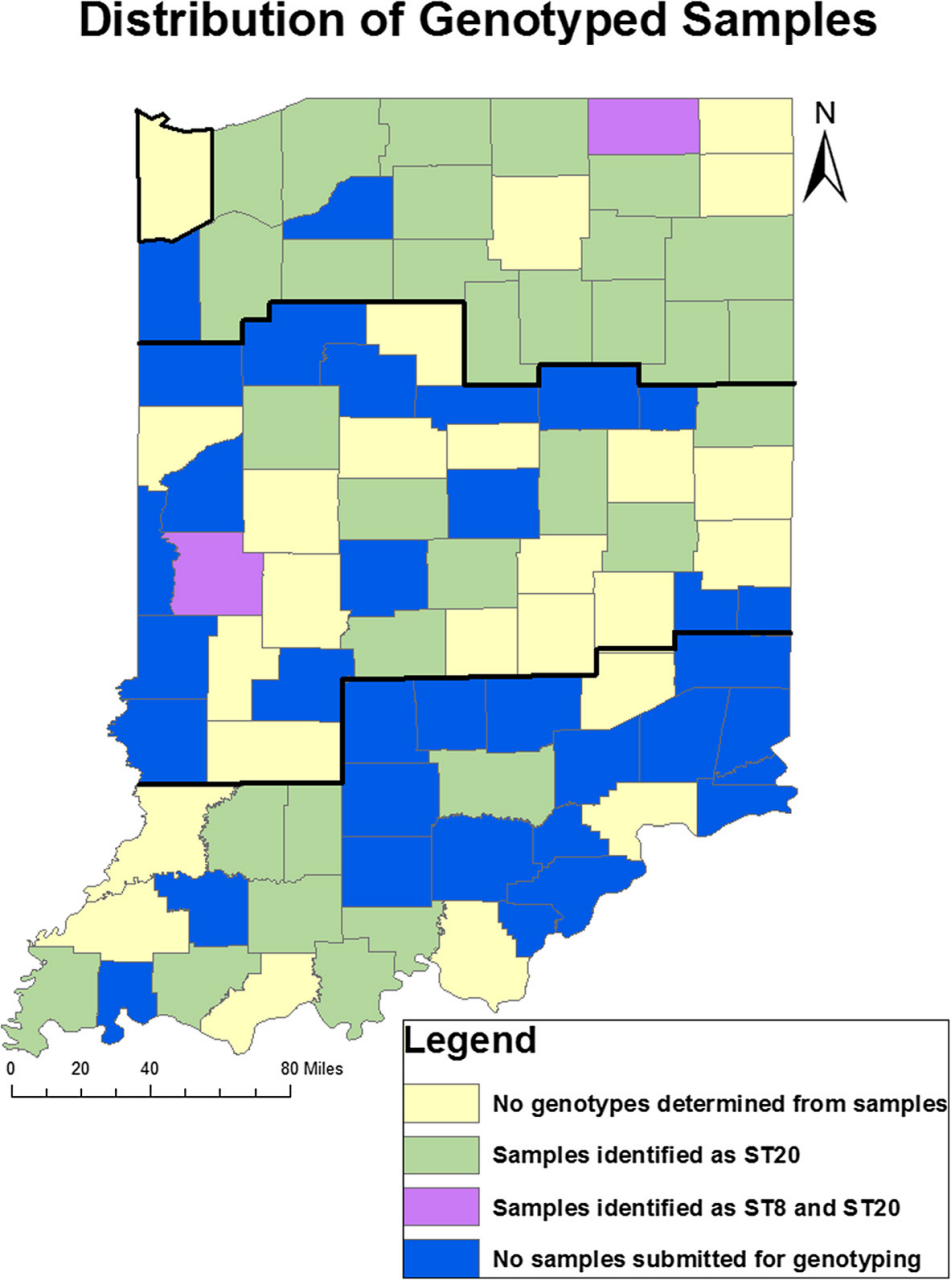
Geographic distribution of DNA samples submitted for *C. burnetii* genotyping. Thick lines indicate regional boundaries as defined by Public Health Preparedness Districts. Lake County, outlined in the Northern region, had no samples testing positive for *C. burnetii* by our cutoff criteria, but a sample demonstrating a CT greater than 36.5 was submitted for genotyping

A selection of 188 DNA samples was submitted for genotyping. Ten samples with CT values between 36.5 and 37 (defined as negative in this study) were included with 178 samples with CT values less than or equal to the positive cut-off value of 36.5. The submitted samples represented 58 of the original 79 counties (Fig. 2). Of these samples, 83 failed to genotype with all assays tested (Table 2). All of the samples with CT values between 36.5 and 37 failed to genotype. *ST20* or *ST8* was positively identified in 74 of the remaining samples (39.4 %). PCR targeting *Cox56bp10* revealed only the derived nucleotide G in 71 samples, identifying *ST20*. PCR targeting *Cox51bp67* revealed only the derived nucleotide C in 2 samples, identifying *ST8*. One sample showed both alleles at *Cox51bp67* (the more sensitive assay for *ST8*) and the derived allele for the *ST20* specific assay, indicating the presence of both *ST20* and *ST8* within that herd. Of the remaining 31 samples, a single genotype could not be positively identified as one or two of the assays failed likely due to low levels of *C. burnetii* DNA. In this group, the presence of *ST20* could be eliminated from 5 samples and *ST8* from 26 samples based upon the amplification of ancestral alleles at those loci.

**Table 2:** Genotyping results based upon SNP analysis

ST results	*ST20*	*ST8*	*ST20* and *ST8*	Exclude *ST20*	Exclude *ST8*	Failure to genotype	Total
Number of samples	71	2	1	5	26	83	188

### Geographic distribution of sequence types

Samples that were definitively sequence typed were present in all regions of Indiana (Fig. 2). *ST20* was present in all regions of Indiana, making up 98 % (95 % confidence interval 90.7–99.9 %) of identifiable ST in the Northern region, 90.9 % (95 % confidence interval 62.7–99.6 %) of identifiable ST in the Central region excluding the farm with a mixture of *ST20* and *ST8* and 100 % (95 % confidence interval 76.2–100 %) of the identifiable ST in the Southern region of the state (Table 3). Two farms with *ST8* (including the farm with the combined result) were located in Parke County in the Central region of Indiana. The third farm where *ST8* was identified was in LaGrange County in the Northern region of Indiana.

**Table 3:** Geographic distribution of sequence types of *C. burnetii*

Region	*ST20*	*ST8*	ST not identified	% ST identified	*ST20* estimated prevalence	95 % C.I.
Northern	50	1	78^b^	60.5 %	93.0 %	90.7–99.9 %
Central	10^a^	1^a^	24	50.0 %	90.9 %^a^	62.7–99.6 %
Southern	11	0	12	47.8 %	100 %	76.2–100 %
Total	71^a^	2^a^	114	64.0 %	97.3 %^a^	91.2–99.5 %

^a^Indicates that the farm with both *ST20* and *ST8* was not included in these calculations

^b^Includes a sample from Lake County that was not identified as positive, but demonstrated a CT greater than the 36.5 cutoff value

## Discussion

The presence of *C. burnetii* in commercial dairy herds in Indiana is not surprising given the ubiquity of the organism in ruminants throughout the world. Samples from Indiana were included in nationwide studies that estimated 90 % prevalence and 77 % prevalence of *C. burnetii* among the samples tested [11, 17]. Outbreaks of Q fever generally occur at a scale smaller than that of the nation [6, 24, 25] and knowledge of prevalence within a given state can impact decisions about resource allocation for surveillance and prevention efforts.

The prevalence estimate of 61 % in this study is slightly lower than that of the national studies [11, 17]. It could be argued that defining a cut-off value for positive samples rather than considering all samples registering CTs as positive may have resulted in an underestimation of prevalence in our study. However, when all samples demonstrating amplification are included as positive in calculations, the prevalence estimate becomes 64.2 % (95 % confidence interval 58.8–69.3 %), essentially no different from the estimate based on the defined cut-off value. Indeed, the confidence intervals for both estimations have considerable overlap indicating that the utilization of a cut-off value in this study did not impact the prevalence estimate.

Identifying the strain or strains of *C. burnetii* present within a herd or geographic location is meaningful in several ways. Recognizing the sequence type present during an outbreak can help to clarify sources and transmission patterns. Although the role of milk and dairy products in human infection is debatable [26, 27], knowing the ST circulating in dairy cattle can help to rule out these products as sources of infection. Understanding which STs dominate in different species can also help to determine if there is transmission of *C. burnetii* between species. This is supported by our findings of ST8 in commercial bulk tank milk samples. However, the lack of information regarding the presence of goats on the farms limits our ability to investigate this conclusion. Certain sequence types have been linked with different clinical presentations of Q fever [21] and thus knowledge of ST may help to predict the clinical course in infected individuals or prepare for the long term repercussions of an outbreak on public health infrastructure. Finally, as *C. burnetii* is considered a Class B bioterror agent, knowledge of the ST circulating within reservoirs at a given location would help to differentiate between a natural outbreak and a bioterror event.

A sequence type was identified in only 39.4 % of the samples in which sequence typing was performed. The target for the initial PCR (*IS1111*) is a multicopy transposon with a highly variable number of copies present per organism [35]. This increases the sensitivity of PCR for detection of *C. burnetii*, but it means that amplification of single copy loci, such as those targeted in SNP analysis, may be more challenging. Indeed, no samples in this study with a cycle threshold greater than or equal to 35 on the initial PCR produced a defined ST.

In our study sample, *ST20* was the main ST identified. This is consistent with previous research indicating that *ST20* is the most common strain of *C. burnetii* circulating in dairy cattle in the United States [23]. The prevalence of *ST20* is greater than 90 % in all geographic regions of Indiana, suggesting a rapid spread of this ST consistent with a model of fast dispersal and persistence proposed by Pearson et al. [23]. However, to improve estimation of ST prevalence at the regional level, a larger sample size would be needed. Increased sensitivity for detecting single copy genes, such as those used for SNP genotyping, would also improve ST identification and prevalence estimation. Interestingly, *ST8* was identified in 3 samples, including one bulk tank sample which contained both *ST8* and *ST20. ST8* has been most commonly associated with goats and this finding suggests possible cross species transmission as well as infection with multiple strains of *C. burnetii* within a herd. In Pearson et al., *ST8* was not found in milk samples from cows [23]. While goat milk samples in that study contained mainly *ST8*, *ST20* was detected in 2 samples [23]. *ST8* has also been associated with the chronic form of Q fever in human patients [21]. Individuals working on farms with *ST8* circulating in the cattle may be at a higher risk for development of the chronic form of Q fever than those working on farms with *ST20* present. Further epidemiologic work is needed to investigate these associations.

Limitations in this study relate to the population of interest, lack of detailed farm level information and lack of specificity analysis for the *IS1111* PCR utilized. Of the 3 species of domestic ruminant reservoirs of *C. burnetii*, only cattle were included in this study. Prevalence and sequence types likely vary between cattle, sheep and goats and only part of the epidemiological system of *C. burnetii* is presented here. In addition, this study focused on herds registered as commercial premises. Commercial dairies represent only half of the premises registered as owning dairy cattle in Indiana (51 % of 2401 premises noted in the 2012 Census of Agriculture). Generalization of these findings to the non-commercial operations should be done with care. An additional concern of utilizing commercial operations is the possibility that individual farms may have more than one bulk tank. Duplicate samples from the same farm may have been included in the study. Upon review of the 1167 samples received, 11 potential duplicates were identified, the majority in Elkhart County. No duplicates were included in the 316 samples selected for *IS1111* testing and genotyping. Although our statewide prevalence estimate was most likely not affected by these duplications, Elkhart County may have been overrepresented in the tested samples. This would affect the prevalence estimate for the Northern region as well as the regional prevalence comparisons. No information was collected about farm level variables such as breed, herd husbandry and the presence of other species present on the farm. This limits our ability explain the source of *ST8* in 3 of the farms. The presence of *ST8* in dairy cattle samples in our study may indicate an association with goats near or on the farm. However, the possibility of cross-contamination between cattle and goat milk at the farm level exists. Obtaining more information about the presence of other dairy species and the management of milk on individual farms would help to address this concern.

Lack of a specificity analysis for the *IS1111* PCR utilized in this study is an additional limitation. PCR targeting the *IS1111* transposon has been evaluated for specificity against several closely and distantly related pathogens in other studies with results indicating that the *IS1111* transposon is a *C. burnetii* specific target for PCR [31, 32]. However, *Coxiella*-like endosymbiotes have been identified in *Amblyomma americanum* [36], the range of which extends into the southern portion of Indiana. It would be of interest to perform sensitivity analyses for the *IS1111* PCR that include these organisms and other non-pathogenic bacteria in addition to the closely related *Legionella* and *Francisella* species.

The presence of a mixed infection within a herd warrants additional investigation with greater attention paid to the presence of other species on the farm. Goals for future research involving *C. burnetii* based on bulk tank or other pooled samples should be to determine how common infection with multiple STs is, potential origins of different STs identified and the implications these have for disease transmission. Additional phylogenetic resolution within *ST20* will be important for identifying patterns of dissemination. Further research is also needed to characterize the STs of *C. burnetii* present in other host species, including human beings, and the patterns of transmission of different STs between species.

## Conclusions

This study is a first step in understanding the epidemiology of *C. burnetii* in Indiana. *C. burnetii* is present in commercial dairy cattle herds throughout the state of Indiana at an estimated prevalence of 61 %. This finding supports the idea that there is variability in *C. burnetii* prevalence at finer geographic levels than the national scale. *ST20* was the most common sequence type present in commercial bulk tank milk samples in Indiana, but *ST8* was also identified. This is the first documentation of *ST8* in milk from cattle in the United States. Although our study supports *ST20* as the dominant ST in cattle, the presence of *ST8* indicates the possibility of transmission of *C. burnetii* between goats and cattle. Identification of both *ST20* and *ST8* within a single herd is an additional unique finding in this study. This supports the idea that *C. burnetii* infection within a species in a given location may be more dynamic than expected and that the dominant ST of *C. burnetii* within a given species may change with time.

## Additional file

Additional file 1:
**Sample selection, county level prevalence and sequence typing data.** This table details the number of samples provided, sample proportional to size calculations, the number of samples tested by county, PHPD and region for *C. burnetii* and the ST of *C. burnetii* identified. Counties labelled with # had samples submitted for sequence typing with CTs above the cutoff for identification as positive in this study. Parke county, labelled with *, was the source of the sample that contained both *ST20* and *ST8*. This sample is included in the total count of *ST20* and *ST8* in this table. Jasper county had samples submitted that were not on the initial documentation and were not included in sample size calculations based on 1385 samples. These samples were accounted for with sample size calculations based on the 1167 samples received. (XLS 44 kb)

## Abbreviations

Bp: Base pair
CI: Confidence interval
ELISA: Enzyme-linked immunosorbent assay
ISBOAH: Indiana state board of animal health
MLVA: Multilocus variability analysis
MST: Multispacer sequence typing
NAHMS: National animal health monitoring system
PCR: Polymerase chain reaction
PHPD: Public health preparedness district
RFLP: Restriction fragment length polymorphism
SNP: Single nucleotide polymorphism
ST: Sequence type

## References

[1] Derrick EH (1937). Q fever, a new fever entity: clinical features, diagnosis and laboratory investigation. Med J Aust.

[2] Hugh-Jones ME, Hubbert WT, Hagstad HV (1995). Zoonoses: recognition, control and prevention.

[3] McQuiston JH, Holman RC, McCall CL, Childs JE, Swerdlow DL, Thompson HA (2006). National surveillance and the epidemiology of human Q fever in the United States, 1978–2004. Am J Trop Med Hyg.

[4] Tissot-Dupont H, Torres S, Nezri M, Raoult D (1999). Hyperendemic focus of Q fever related to sheep and wind. Am J Epidemiol.

[5] Tissot-Dupont H, Amadei M-A, Nezri M, Raoult D (2004). Wind in November, Q fever in December. Emerg Infect Dis.

[6] Schimmer B, ter Schegget R, Wegdam M, Zuchner L, de Bruin A, Schneeberger PM (2010). The use of a geographic information system to identify a dairy goat farm as the most likely source of an urban Q-fever outbreak. BMC Infect Dis.

[7] Oyston PC, Davies C (2011). Q fever: the neglected biothreat agent. J Med Microbiol.

[8] Norlander L (2000). Q fever epidemiology and pathogenesis. Microbes Infect.

[9] Arricau-Bouvery N, Rodolakis A (2005). Is Q fever an emerging or reemerging zoonosis?. Vet Res.

[10] Rodolakis A, Berri M, Hechard C, Caudron C, Souriau A, Boudier CC (2007). Comparison of *Coxiella burnetii* shedding in milk of dairy bovine, caprine, and ovine herds. J Dairy Sci.

[11] Kim SG, Kim EH, Lafferty CJ, Dubovi E (2005). *Coxiella burnetii* in bulk tank milk samples, United States. Emerg Infect Dis.

[12] Guatteo R, Beaudeau F, Joly A, Seegers H (2007). Assessing the within herd prevalence of Coxiella burnetii milk-shedder cows using a real-time PCR applied to bulk tank milk. Zoonoses Public Health.

[13] Guatteo R, Beaudeau F, Joly A, Seegers H (2007). *Coxiella burnetii* shedding by dairy cows. Vet Res.

[14] Garcia-Perez AL, Astobiza I, Barandika JF, Atxaerandio R, Hurtado A, Juste RA (2009). Investigation of *Coxiella burnetii* occurrence in dairy sheep flocks by bulk tank milk analysis and antibody level determination. J Dairy Sci.

[15] Angen O, Stahle M, Agerholm JS, Christoffersen AB, Agger JF (2011). Dynamics of the relationship between the presence of *Coxiella burnetii* DNA, antibodies and intrinsic variables in cow milk and bulk tank milk from Danish dairy cattle. J Dairy Sci.

[16] Rahimi E, Ameri M, Karim G, Doosti A (2011). Prevalence of *Coxiella burnetii* in bulk milk samples from dairy bovine, ovine, caprine, and camel herds in Iran as determined by polymerase chain reaction. Foodborne Pathog Dis.

[17] APHIS Veterinary Service Centers for Epidemiology and Animal Health (2011). Prevalence of *Coxiella burnetii* in bulk-tank milk on U.S. dairy operations, 2007.

[18] Jager C, Willems H, Thiele D, Baljer G (1998). Molecular characterization of *Coxiella burnetii* isolates. Epidemiol Infect.

[19] Roest HIJ, Ruuls RC, Tilburg JJHC, Nabuurs-Franssen MH, Klaassen CHW, Vellema P (2011). Molecular epidemiology of *Coxiella burnetii* from ruminants in Q fever outbreak, the Netherlands. Emerg Infect Dis.

[20] Frangoulidis D, Walter MC, Antwerpen M, Zimmerman P, Janowetz B, Alex M (2014). Molecular analysis of *Coxiella burnetii* in Germany reveals evolution of unique clonal clusters. Int J Med Microbiol.

[21] Glazunova O, Roux V, Freylikman O, Sekeyova Z, Fournous G, Tyczka J (2005). *Coxiella burnetii* genotyping. Emerg Infect Dis.

[22] Hornstra HM, Priestley RA, Georgia SM, Kachur S, Birdsell DN, Hilsabeck R (2011). Rapid typing of *Coxiella burnetii*. PLoS One.

[23] Pearson T, Hornstra HM, Hilsabeck R, Gates LT, Olivas SM, Birdsell DM (2014). High prevalence and two dominant host-specific genotypes of *Coxiella burnetii* in U.S. milk. BMC MIcrobiol.

[24] Signs KA, Stobierski MG, Gandhi TN (2012). Q fever cluster among raw milk drinkers in Michigan, 2011. Clin Infect Dis.

[25] Bjork A, Marsden-Haug N, Nett RJ, Kersh GJ, Nicholson W, Gibson D (2014). First reported multistate human Q fever outbreak in the United States, 2011. Vector-Borne Zoonotic Dis.

[26] Cerf O, Condron R (2006). *Coxiella burnetii* and milk pasteurization: An early application of the precautionary principle?. Epidemiol Infect.

[27] Gale P, Kelly L, Mearns R, Duggan J, Snary EL (2015). Q fever through consumption of unpasteurised milk and milk products - a risk profile and exposure assessment. J Appl Microbiol.

[28] United States Department of Agriculture (2014). Census of agriculture 2012.

[29] United States Department of Agriculture (2014). Census of agriculture 2012.

[30] United States Department of Agriculture (2014). Census of agriculture 2012.

[31] Panning M, Kilwinski J, Greiner-Fischer S, Peters M, Kramme S, Frangoulidis D (2008). High throughput detection of Coxiella burnetii by real-time PCR with internal control system and automated DNA preparation. BMC Microbiol.

[32] Klee SR, Tyczka J, Ellerbrok H, Franz T, Linke S, Baljer G (2006). Highly sensitive real-time PCR for specific detection and quantification of *Coxiella burnetii*. BMC Microbiol.

[33] Altschul SF, Gish W, Miller W, Myers EW, Lipman DJ (1990). Basic local alignment search tool. J Mol Biol.

[34] Dean AG, Sullivan KM, Soe MM (2014). OpenEpi: Open source epidemiologic statistics for public health.

[35] Klee SR, Ellerbrok H, Tyczka J, Franz T, Appel B (2006). Evaluation of a real-time PCR assay to detect *Coxiella burnetii*. Ann N Y Acad Sci.

[36] Klyachko O, Stein BD, Grindle N, Clay K, Fuqua C (2007). Localization and visualization of a *Coxiella-*type symbiont within the Lone Star Tick, *Amblyomma americanum*. Appl Environ Microbiol.

[37] United States Department of Agriculture (2014). Census of agriculture 2012.

